# Effect of Ethanol Consumption on the Accuracy of a Glucose Oxidase-Based Subcutaneous Glucose Sensor in Subjects with Type 1 Diabetes

**DOI:** 10.3390/s22093101

**Published:** 2022-04-19

**Authors:** Vanessa Moscardó, Alia Garcia, Jorge Bondia, Julián Diaz, Agustín Ramos-Prol, Paolo Rossetti

**Affiliations:** 1 Facultad de Ciencia y Tecnología, Universidad Internacional de Valencia, 46002 Valencia, Spain; 2 Department of Endocrinology, University Hospital of La Ribera, 46600 Alzira, Spain; garcia_alicasa@gva.es; 3 Instituto Universitario de Automática e Informática Industrial, Universitat Politècnica de València, 46022 Valencia, Spain; jbondia@isa.upv.es; 4 Centro de Investigación Biomédica en Red de Diabetes y Enfermedades Metabólicas Asociadas (CIBERDEM)—Grupo CB17/08/00004, Instituto de Salud Carlos III, 41092 Madrid, Spain; rossetti_pao@gva.es; 5 Hospital Francesc de Borja, 46702 Gandia, Spain; diaz_jul@gva.es (J.D.); agustinprol@hotmail.com (A.R.-P.); 6 Department of Endocrinology, Hospital Universitari i Politècnic La Fe, 46026 Valencia, Spain

**Keywords:** type 1 diabetes, alcohol, glucose-sensor accuracy, glucose oxidase-based sensors, continuous glucose monitors

## Abstract

Continuous glucose monitors (CGM) have improved the management of patients with type 1 diabetes (T1D), with glucose oxidase (GOx)-based sensors being the most used. However, they are potentially subject to both electrochemical and enzymatic interferences, including those related to changes of pH. The objective of this study is to investigate the effect of ethanol, given as beer along with a mixed meal, on the accuracy of a commercial GOx-CGM. Data from 12 T1D participants in a randomized crossover trial to evaluate the effect of meal composition and alcohol consumption on postprandial glucose concentration were used. Absolute error (AE) and mean absolute relative difference (MARD) were calculated. The differences between the alcohol and nonalcohol scenarios were assessed using the Mann–Whitney U and Wilcoxon signed-rank tests. The AE in the alcohol study was low, but significantly greater as compared to the study without alcohol (*p*-value = 0.0418). The MARD was numerically but not significantly greater. However, both variables were greater at pH < 7.36 and significantly affected by time only in the alcohol arm. In T1D, alcohol consumption affects the accuracy of a GOx-CGM. This effect could be at least partially related to the ethanol-induced changes in pH.

## 1. Introduction

Continuous glucose monitors (CGM), either in real time (rtCGM) or intermittent scanning (isCGM), have substantially improved the management of patients with type 1 diabetes (T1D), allowing for better metabolic control and quality of life [1].

The current market of CGM is dominated by the electrochemical, enzymatic-based technology, with the single exception of the Eversense (Senseonics, Germantown, MD, USA) [2]. All of them measure glucose concentration in the interstitial fluid (ISF) through glucose oxidase (GOx). However, currently available rtCGMs use first-generation sensors, which utilize oxygen as an electron acceptor, whereas isCGMs use second-generation technology where an artificial electron mediator is used to transfer electrons to the sensor’s electrode, making it independent of fluctuations in local tissue oxygen and mitigating interferences from substances other than glucose [3]. Nevertheless, all of the enzyme-based sensors are potentially subject to both electrochemical and enzymatic interferences [4], including those related to changes of pH, which is known to vary more in the ISF (6.6–7.6) than in plasma [5]. Indeed, the pH is known to affect the anomeric ratio between ß-D-Glucose (the predominant form of glucose in body fluids) and α-D-Glucose: basic conditions prefer the ß-D-glucose anomer, and acidic conditions the α-D form, the interconversion between the two anomers being slow (hours to reach the equilibrium). Therefore, given that the GOx is specific for the ß form, fluctuations in the ISF’s pH may impair the accuracy and reliability of CGM [5]. An indirect datum suggesting the possible effect of pH on GOx-based CGM is the finding of lower accuracy in diabetic ketoacidosis [6]. However, to the best of our knowledge, no formal analysis of the relationship between pH and CGM accuracy under condition of DKA in humans has been published. Nevertheless, one of the recognized limitations of CGM implementation in the hospital setting is the unknown accuracy under conditions of metabolic acidosis [7] although factors other than pH (such as hypovolemia with peripheral tissue hypoperfusion) may well affect glucose-sensor readings in these cases.

Despite the widespread consumption of alcoholic drinks among patients with T1D (not different from the general population) [8,9], and the increasing importance acquired by CGM in glucose management (with isCGM supplanting capillary testing and rtCGM being implemented in closed-loop systems), there is a scarcity of clinical data about the effect of ethanol on the accuracy and reliability of CGM. Although manufacturers do not declare direct interference of ethanol on their CGM (unlike other small molecules such as ascorbic acid and acetaminophen, which interfere with some of commercially available CGM being oxidized against the sensor’s electrode domain), indirect mechanism may intervene in vivo. Indeed, the only published study that evaluated the effect of ethanol in humans, given as wine in healthy subjects, on a GOx-based CGM [10], demonstrated significant interference. Surprisingly, no data are available in subjects with T1D, despite the known stimulating effect of alcohol on lactate generation (inhibition of the peripheral conversion of lactate into pyruvate) and ketogenesis (conversion of the ethanol-derived acetoacetate into β-OH-Butyrate), leading to significant variations of pH [11].

The objective of the present study was to investigate the effect of ethanol, given as beer along with a mixed meal, on the accuracy of a commercial GOx-based CGM.

## 2. Materials and Methods

### 2.1. Study Design

The study design and methods have been described in detail elsewhere [12]. Briefly, twelve subjects with T1D on a basal-bolus insulin regimen and free of significant micro- and macrovascular complications were recruited among those attending to the outpatient clinic of the Francesc de Borja Hospital, Gandía, Spain. They were studied after approval of the Ethical Committee of the Hospital Clínic Universitari de València and giving informed written consent.

The study had a randomized, crossover, open design, with each subject receiving a mixed meal on three different occasions at two-week intervals. On two occasions the solid component of the meal was identical, but patients received a different drink: on one occasion, beer at a volume calculated to administrate 0.7 g of alcohol per kg of weight (High-Protein High-Fat with alcohol—HPHF-A); on the other occasion, a nearly identical volume of a nonalcoholic beer (High-Protein High-Fat without alcohol—HPHF-W). The third mixed meal had the same amount of nonalcoholic beer given in the HPHF-W study, but lower content of fats and proteins (Low-Protein Low-Fat study, without alcohol—LPLF-W).

The subjects came to the hospital at 8 a.m. in a fasting state. Two venous lines were cannulated: the cubital vein for insulin or glucose infusion, if needed, to standardize blood-glucose concentration around 100 mg/dL prior to the meal test (at 10:30 a.m.), and a distal vein of the contralateral arm for arterialized venous blood sampling. Plasma glucose was measured every 5–15 min in arterialized venous blood, centrifugated immediately after the extraction and analyzed in duplicate using a YSI 2300 STAT Plus analyzer (Yellow Springs Instruments, Yellow Springs, OH, USA). Interstitial glucose was measured with a commercial GOX-based CGM (Dexcom G5, Dexcom, San Diego, CA, USA), which was inserted the day prior to the mixed-meal study and calibrated on the morning of the test with capillary blood glucose (always at the same time and with the same glucometer).

Blood samples for determination of ethanol, lactate, and pH were drawn from 30 min before mealtime at 10:00 h (time −30 min) at 30 min intervals during the first two hours after meal (time +120 min), and then every 60 min until the termination of the study at time +360 min. Ethanol was measured by an enzymatic method with alcohol dehydrogenase, pH, and lactate by an ion-selective electrode potentiometry method (Roche Diagnostics SLU, Sant Cugat del Valles, Spain).

### 2.2. Data Processing

Data from the LPLF-W study were not included in the analysis to avoid any possible confounding factor related to differences in meal composition (other than ethanol). Then, all patients’ recordings of plasma glucose (PG), plasma alcohol (PA), plasma pH concentration, and the CGM values were considered, taking into account the branch of study (HPHF-W, HPHF-A).

Sensor accuracy is usually given by the absolute error (AE) and the mean absolute relative difference (MARD). MARD is used since it is the most widely used measure to assess the measurement quality of CGM systems due to its simplicity, despite its limitations [13]. Both metrics are calculated using the PG and CGM data. On the study recordings, the sampling period of PG was lower than the CGM; thus, a linear interpolation of the CGM was carried out to have a CGM sample coincident with each sampling time of PG. From PG and interpolated CGM values, AE and MARD were calculated for each considered instant, according to the formulations in Table 1.

On the other hand, to increase the sensitivity of the analysis and to also consider the potential indirect effect of ethanol on CGM through changes in pH, two new variables were created to make a binary classification of the pH and PA levels based on the following thresholds (Table 1): 0.3 mg/mL for ethanol (the legal upper limit allowed for professional drivers in Spain), a threshold easily achieved with 1–2 units of alcohol and below which a significant metabolic effect is not likely; and 7.36 for pH, as it is the lower limit of normality.

### 2.3. Statistical Analysis

Statistical analysis was carried out using R 4.1.0 and several procedures were applied to the data, depending on the aim.

Firstly, the sensor accuracy, given by the AE and MARD, during the HPHF-A study was compared with the one during the HPHF-W study. A D’Agostino’s K-squared test was performed to assess the normality of AE and MARD data. After that, due to the non-normality of the data (See Supplementary Materials: Figures S1 and S2), nonparametric tests (Mann–Whitney U test and Wilcoxon signed-rank test, for nonpaired and paired comparisons, respectively) were used to assess if there were statistically significant differences between the HPHF-A and the HPHF-W study. We also evaluated the effect of time on accuracy, and the interaction between study and time, considering a 30 min sampling time to limit the number of multiple comparisons. The significance level (α) considered was 0.05; a Bonferroni correction was applied when post hoc multiple comparisons were performed on significant terms and interactions.

Secondly, the relationship between alcohol and pH levels and the sensor accuracy was evaluated. To this end, information about PA and pH besides the AE and MARD variables was included in the analysis by means of the transformed categorical variables, PA_Level and pH_Level (see Table 1). Again, a nonparametric test was computed due to the non-normality of the data (Mann–Whitney U test), with significance set at alfa 0.05.

## 3. Results

This section presents the main results achieved through the application of the methodology described above. The analysis of the outputs obtained is divided into two parts to make their presentation clearer. Importantly, PG values of the HPHF-A and HPHF-W studies were very similar [12].

### 3.1. Evaluation of the Sensor-Accuracy Differences between Studies

The AE and MARD for each patient and study were calculated and the main statistical indicators of both metrics were obtained, as shown in Table 2. The AE of the entire postprandial period in the HPHF-A study was low, but significantly greater as compared to the study without alcohol (*p*-value = 0.0418). The median MARD in case of alcohol consumption was numerically greater but not significantly different from the HFHP-W study.

The paired comparison by means of the Wilcoxon Test (Table 3) also supports the statistically significant difference between the AE during the study with alcohol and without it (*p*-value < 0.001). The AE mean difference between the HPHFA and HPHF-W studies was 1.71, 95%CI [1.03; 2.39] mg/dL and the median 2.35 [1.72; 3.57] mg/dL. These values could be not clinically relevant; nevertheless, the fact that the difference was statistically significant demonstrates that the alcohol concentration may affect the sensor accuracy in terms of absolute error. Regarding the MARD, the mean difference between studies was 1.22% with the 95%CI [0.76; 1.68] and the difference of medians 1.03 [0.94; 2.58]%. These differences are relatively small and not statistically significant (*p* = 0.08), but considering this discrepancy might be interesting, depending on the error range of the used sensor.

**Figure 1:**
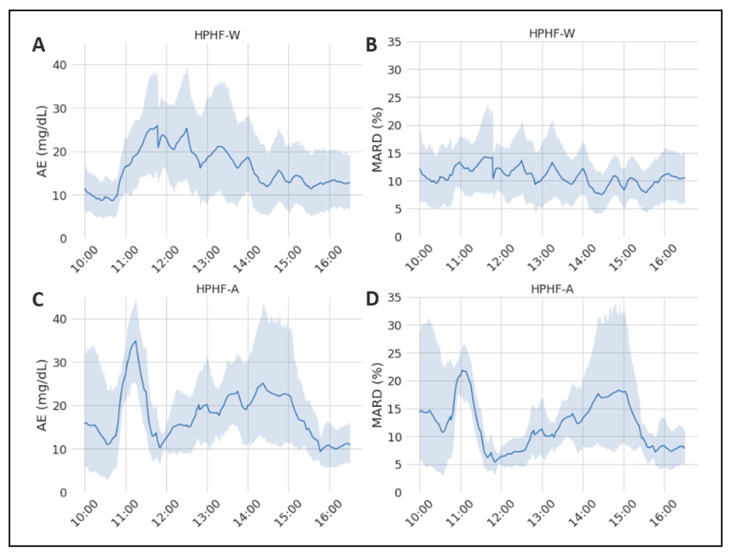
Representation of the mean values of the considered sensor-accuracy metrics during the study duration. The grey area represents the 95% CI. (**A**) Evolution of the AE during the HPHF-W study; (**B**) evolution of the MARD during the HPHF-W study; (**C**) evolution of the AE during the HPHF-A study; (**D**) evolution of the MARD during the HPHF-A study.

When analyzing the performance of the EA and MARD across time (Figure 1) in the two studies of interest, we found no significant effect of time under the HPHF-W condition (*p* = 0.160 for AE and 0.887 for MARD). In contrast, both the AE and the MARD changed significantly over time following alcohol ingestion (*p* value for AE and MARD respectively 0.0059 and 0.0015). In particular, in the HPHF-W study the AE increased (nonsignificantly) after the meal, as expected under conditions of rapid glycemic variations (due to the delay between plasma and interstitial glucose), coming back to basal values in the late postprandial phase when plasma glucose was more stable. In contrast, the effect of alcohol appeared complex, as in the HPHF-A study the AE increased faster and to a greater extent shortly after the meal (AE at 11 h significantly different from the other time points but 11:30, 13, 13:30 h), decreased rapidly and consistently in all subjects in the 90–180 min postprandial period, to increase again significantly above baseline (at time 14–14:30 h (Figure 1A,C). Accordingly, the MARD was quite constant in the HPHF-W study, while exhibited wide variations in the HPHF-A study being significantly greater than the rest at 11 h, lower at 12 h as compared with values at 11 h and 14–15 h and greater than basal around 14 h (Figure 1B,D).

**Figure 2:**
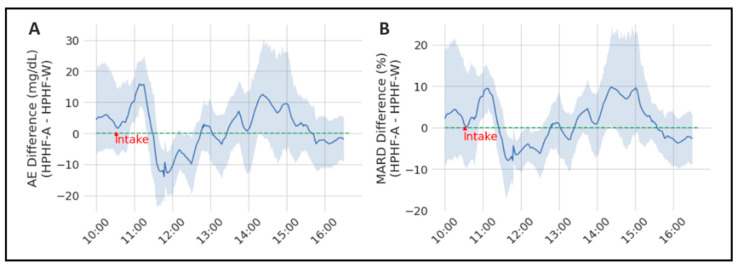
Representation of the mean AE (panel (**A**)) and MARD differences (panel (**B**)) between both considered studies (HPHF-A and HPHF-W). The grey area represents the 95% CI.

For the sake of clarity, the difference of AE and MARD between both studies was calculated and is illustrated in Figure 2 (positive values of the difference means that the error of the study with alcohol is greater), which shows the time-variant effect of alcohol on CGM accuracy. The interaction between study and time was significant for both the AE (*p* = 0.0089) and the MARD (*p* = 0.0459), with post hoc comparisons showing significant differences between HPHP-A and HPHF-W at time 11 h, 12 h and 14:30 h for the former, and at 11 h for the latter.

### 3.2. Evaluation of the Relationship between Alcohol and pH Levels and Sensor Accuracy

The outputs of this part are in line with the previous ones, with greater plasma alcohol concentration associated with lower CGM accuracy. Moreover, Table 4 summarizes the main statistical indicators of AE and MARD, aggregated by the low and high levels of concentration for each variable considered to represent the categorical values of PA and pH concentration (i.e., PA_Level and pH_Level).

The AE in the low-level concentrations of pH was statistically different from the high-level concentrations, with the AE being higher when pH concentrations are low. This behavior is in accordance with the results obtained with PA_Level, the AE being significantly greater when PA concentrations are high.

Regarding the MARD, it was numerically greater with plasma alcohol above 0.3 mg/mL, and at lower pH concentrations, but the differences did not reach statistical significance (Table 4).

## 4. Discussion

This study demonstrates that alcohol consumption (beer) in the context of a mixed meal in subjects with T1D, significantly affects the accuracy of a GOx-based rtCGM and suggests that this effect is at least partially related to the ethanol-induced changes in pH. Although the mean magnitude of the effect over a 6 h postprandial period is small, ethanol induces larger variations over time of both the AE and MARD, the latter being up to 10% greater in the HPHF-A study than the HPHF-W condition at some time points (Figure 2). Surprisingly, ethanol also appears to transiently improve the accuracy at some other time points (roughly 1.5 h post-ingestion). This effect is not likely to be due to chance, as it was very consistent among subjects (as shown by the narrowing of the 95%CI, with significant post hoc comparisons), and explains the small impact on the mean AE and MARD. Although in our study we did not measure interstitial levels of ethanol, we used an oral dose similar to others [10] who did, and like them, we found a significant effect on the CGM signal. The interference seems to be present starting from relatively low plasma ethanol concentrations, as the AE of those samples with ethanol > 0.3 mg/mL (the median concentration in our study was 0.38 mg/mL, with p10 and p90 respectively 0.32 and 0.45 mg/mL) was 6 mg/dL greater than the rest. However, the effect was small in absolute terms.

After its ingestion, ethanol diffuses rapidly throughout the body [14], being rapidly detectable in the ISF [10], making a direct effect of ethanol on the glucose sensor through electrochemical interference theoretically possible. However, this is not likely due to the improvements of manufacturing of the sensors’ interference-rejection domains, which prevent small molecules with redox potential (such as ascorbic acid, acetaminophen…) from reaching the electrode domains [2]. Moreover, no such an interference has been mentioned in recent reviews on the topic [15,16,17]. One study investigated, in vitro, the interference of six sugars and six alcohols (but not ethanol) on three commercial GOx-based CGM, excluding any impact of the latter on sensor accuracy [4]. Nevertheless, the profound ethanol-induced metabolic alterations may change the relationship between plasma and interstitial glucose. Indeed, its oxidation induces a state of insulin resistance as its main product, acetate, is converted into acetyl CoA that enters de Krebs cycle, limiting glucose and FFA oxidation. Moreover, by increasing the intramitochondrial NADH/NAD+ ratio, it decreases the free AMP concentrations inhibiting the AMPK activity resulting in downregulation of glucose transporters, as well as in the subcutaneous tissue [14]. However, to the best of our knowledge, whether ethanol affects the glucose dynamic between plasma and the subcutaneous ISF has not been investigated.

On the other hand, ethanol may affect CGM signal indirectly, through changes in interstitial fluid pH. Indeed, changes in pH are also known to affect the rate of glucose oxidation by GOx (and therefore the sensor’s signal) in the range that can be observed in the interstitial fluid [18]. Additionally, as already mentioned, pH affects the interconversion between α-D and β-D glucose, altering the anomeric ratio of glucose molecules in body fluids including in the interstitial space [5]. This is relevant for those detection techniques that are anomer-specific (as is the case for GOx) potentially leading to errors, given that the timescale for anomer equilibration (hours) is significantly longer than the time required to perform the measurements (seconds). However, due to the obvious barriers to study the effect of different interstitial pH values on CGM in clinical studies, only a few data on the topic are available, mainly in the intensive care setting (ICU). One study in a pediatric ICU found no relationship between pH (and lactate) levels and the accuracy of a commercial GOx-based CGM [19]. However, findings in that setting should be interpreted with caution, as many other factors, such as peripheral-tissue hypoperfusion, may affect (in different directions) the sensor signal, masking the effect of pH. Indeed, studies performed in ICUs have generally—but not consistently—found lower CGM performance [20], with some notable exceptions such as better accuracy in septic shock [21]. In our study [12], plasma pH was significantly lower following alcohol ingestion as compared to the study without alcohol (HPHF-A median pH 7.36, range 7.31–7.43; HPHF-W median 7.38, range 7.34–7.45) and the accuracy appeared reduced, with pH levels < 7.36 associated with an AE about 4 mg/dL and a MARD a 1% greater than with pH equal or above that threshold. Again, the effect was small in absolute terms.

Our work has some strengths. It is the first study that has investigated the effect of ethanol on the accuracy of a CGM in subjects with T1D, in the post prandial state (i.e., the most common way of alcohol ingestion, with a mixed meal). Importantly, the study had a crossover design and the macronutritional composition of the meal was nearly identical in both arms, eliminating any possible confounding factor. Secondly, we measured plasma alcohol concentration and pH systematically, with a frequency apt to detect a transient effect on accuracy. However, it has also some limitations. First of all, the analysis was limited only to the postprandial state, when large and fast variations of blood glucose are expected, likely increasing the observed MARD and error due to the physiological and device-related delays [22]; this in turn may have masked, at least partially, the effect of ethanol, which we have shown (as expected) to be time-variant in vivo (following its absorption, distribution, and metabolism). Additionally, interstitial ethanol and pH were not measured, and this could be particularly relevant for the latter. Indeed, variations of pH are expected to be larger in the ISF than in plasma (for the scarcity of buffer compounds) and therefore using plasma measurements (where variations are buffered) may have given a weaker-than-real association between pH and accuracy. Finally, the sample size was relatively small, not allowing to fully catch relatively small but significant differences in the MARD.

In conclusion, ethanol ingestion with a mixed meal appears to affect CGM accuracy in people with T1D. The interference pattern is complex, and likely driven by different ethanol-induced phenomena, which probably act in different directions. Larger studies are needed to better define the relationship between ethanol metabolism and the changes in the CGM signal, and to elucidate the mechanisms responsible for those changes.

## Figures and Tables

**Table 1:** Set of variables used for assessing the effect of the alcohol on the sensor accuracy.

Type of Variable	Variable	Meaning
Measured variables	PG	Plasma glucose recordings Sampling period: each 15 min
	CGM	Continuous glucose-monitoring measurements Sampling period: each 5 min
	PA	Plasma alcohol recordings Sampling period: Each 30 min until two hours after meal, then each 60 min
	pH	Plasma pH recordings Sampling period: Each 30 min until two hours after meal, then each 60 min
	study	Type of study Categorical values: {HPHF-A, HPHF-W, LPLF-W}
Calculatedvariables	AE	Absolute error calculated as: AE=|CGM−PG|
	MARD	Mean absolute relative difference calculated as: $\mathrm{MARD}=\frac{\left|\mathrm{CGM}-\mathrm{PG}\right|}{\mathrm{PG}}\cdot 100$
	PA_Level	Category of plasma alcohol level determined as: $\mathrm{PA}\_\mathrm{Level}=\{\begin{array}{cc} ‘\mathrm{Low}’ & \mathrm{PA}\;<0.3\;\mathrm{mg}/\mathrm{mL}\\ ‘\mathrm{High}’ & \mathrm{Otherwise} \end{array}$
	pH_Level	Category of plasma pH level determined as: $\mathrm{pH}\_\mathrm{Level}=\{\begin{array}{cc} ‘\mathrm{Low}’ & \mathrm{pH}\;<7.36\\ ‘\mathrm{High}’ & \mathrm{Otherwise} \end{array}$

**Table 2:** Statistical main indicators for AE and MARD by study, besides the results of the comparison Mann–Whitney U test between studies for the variable of study.

Metrics	Study	N	Mean (Std)	Median [IQR]	Range	Mann-Whitney U Test
AE	HPHF-A	312	17.93 (17.839)	13.15 [6.55; 22.50]	[0.00; 105.0]	0.0418 *
	HPHF-W	312	16.21 (15.988)	10.80 [4.925; 22.425]	[0.00; 90.6]	
MARD	HPHF-A	312	12.239 (13.635)	9.177 [5.132; 14.11]	[0.00; 96.82]	0.248
	HPHF-W	312	11.018 (9.152)	8.149 [4.184; 16.02]	[0.00; 49.06]	

(*) Statistically significant (α = 0.05).

**Table 3:** Statistical main indicators for AE and MARD by study, besides of the results of the comparison Mann–Whitney U test between studies for the variable of study.

Metrics	Difference (HPHF-A—HPHF-W) Mean [CI95%]	Wilcoxon Paired Test (*p*-Value)
AE	1.71 [1.03; 2.39]	<0.001 *
MARD	1.22 [0.76; 1.68]	0.080

(*) Statistically significant (α = 0.05).

**Table 4:** Statistical main indicators for pH_L1, pH_L2, PA_L1 and PA_L1 by Level value (Low and High), besides of the Mann–Whitney U test results for each variable of study.

		Level Value	N	Mean (Std)	Median[IQR]	Range	Mann–Whitney U Test
AE	pH_Level	Low	83	17.63 (13.37)	15.40 [8.45; 20.75]	[0.00; 58.80]	0.028 *
		High	277	16.08 (16.19)	11.80 [5.13; 22.00]	[0.00; 104.50]	
	PA_Level	Low	277	15.29 (15.09)	11.15 [5.05; 20.20]	[0.00; 99.40]	<0.001 *
		High	83	20.25 (16.65)	17.10 [8.80; 24.68]	[0.50; 104.50]	
MARD	pH_Level	Low	83	12.04 (10.53)	9.48 [5.64; 15.19]	[0.00; 78.18]	0.091
		High	277	11.47 (11.61)	8.49 [3.70; 15.71]	[0.00; 91.19]	
	PA_Level	Low	277	11.22 (10.77)	8.58 [3.91; 15.03]	[0.00; 91.19]	0.097
		High	83	12.85 (13.15)	9.24 [5.96; 16.45]	[0.00; 84.94]	

(*) Statistically significant (α = 0.05).

## Data Availability

The data are available on request from the authors.

## Supplementary Materials

The following supporting information can be downloaded at: https://www.mdpi.com/article/10.3390/s22093101/s1, Figure S1: Probability density (left) and Quantile-Quantile (Q-Q) plot of Absolute Error, AE (right). It can be observed that data is non-normally distributed; Figure S2: Probability density (left) and Quantile-Quantile (Q-Q) plot of MARD (right). It can be observed that data is non-normally distributed.
