# Supplementary material for: Effect of Ethanol Consumption on the Accuracy of a Glucose Oxidase-Based Subcutaneous Glucose Sensor in Subjects with Type 1 Diabetes
Source: Sensors (Basel). 2022 Apr 19;22(9):3101. doi: 10.3390/s22093101 (PMC9104985; doi:10.3390/s22093101)
Supplement: Supplementary file 1 [file sensors-22-03101-s001.zip › sensors-1659899-supplementary.pdf]

Supplementary Materials

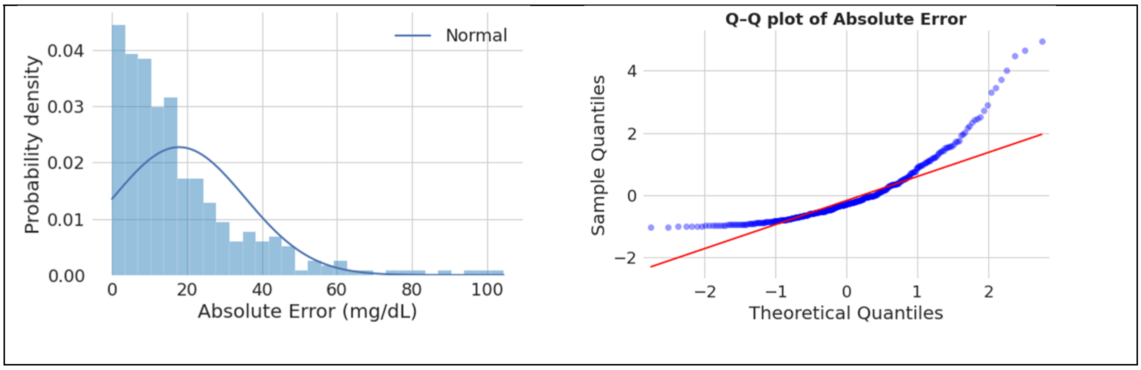

Figure S1. Probability density (left) and Quantile-Quantile (Q-Q) plot of Absolute Error, AE (right). It can be observed that data is non-normally distributed.

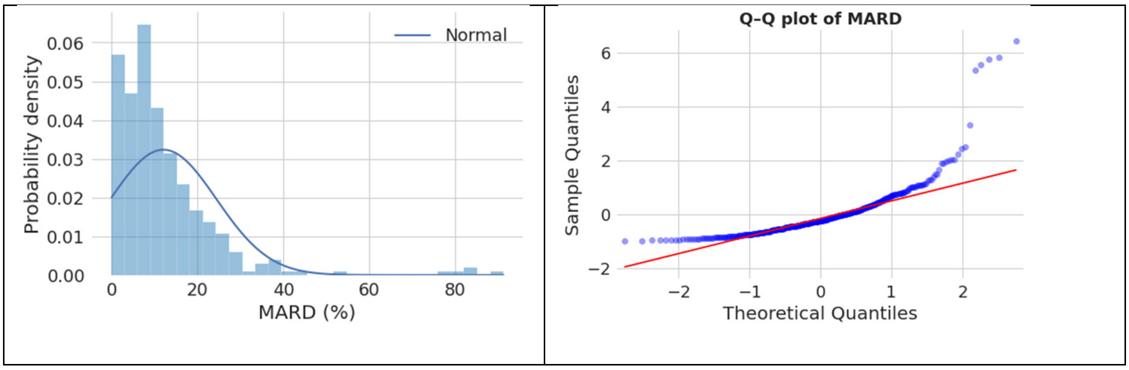

Figure S2. Probability density (left) and Quantile-Quantile (Q-Q) plot of MARD (right). It can be observed that data is non-normally distributed.
